# Navigating Diagnostic Challenges in Ectopic Parathyroid Adenomas: A Case Report

**DOI:** 10.7759/cureus.68637

**Published:** 2024-09-04

**Authors:** Henrick Ryan C Fong, Mihail Zilbermint

**Affiliations:** 1 Internal Medicine, Ascension Saint Agnes Hospital, Baltimore, USA; 2 Endocrinology, Diabetes and Metabolism, Suburban Hospital, Bethesda, USA

**Keywords:** hyperparathyroid, hyperparathyroid-induced hypercalcemia, high pth, anterior mediastinal mass, spect imaging, parathyroid gland adenoma

## Abstract

Ectopic parathyroid adenomas pose significant diagnostic and therapeutic challenges due to their atypical locations outside the usual anatomical boundaries of the parathyroid glands. These adenomas, which represent a small percentage of primary hyperparathyroidism cases, are often found in areas such as the mediastinum, thymus, or retroesophageal space. Their ectopic nature complicates diagnosis, as traditional neck imaging techniques may fail to localize these glands. We present the case of a 27-year-old female who initially presented with nausea, vomiting, severe hypercalcemia, and elevated parathyroid hormone (PTH) levels. Despite being advised to consult an endocrinologist, she experienced difficulty scheduling an appointment. Due to persistent symptoms and laboratory abnormalities, she was subsequently admitted to the hospital. Initial neck imaging failed to identify the parathyroid adenoma. However, subsequent imaging, including parathyroid scintigraphy, revealed an ectopic parathyroid adenoma located in the mediastinum. The patient underwent a successful robotically assisted thymectomy, guided by intraoperative PTH monitoring, which resulted in the resolution of hypercalcemia and normalization of PTH levels. This case underscores the importance of a comprehensive diagnostic approach when dealing with ectopic parathyroid adenomas. Parathyroid scintigraphy, in particular, proves to be a critical tool due to its high sensitivity in detecting ectopic glands. Moreover, our findings emphasize the need for a high index of suspicion for ectopic parathyroid adenomas, especially when conventional neck imaging is inconclusive in cases of hyperparathyroidism. Timely and accurate diagnosis is essential for facilitating precise surgical intervention, ultimately leading to improved patient outcomes.

## Introduction

Ectopic parathyroid adenomas represent a rare but clinically significant subset of parathyroid disorders, often presenting diagnostic challenges due to their anomalous anatomical locations [1]. While most parathyroid adenomas are found within the cervical region, ectopic adenomas can occur anywhere along the descent pathway of the embryonic parathyroid glands, including the mediastinum [2]. The mediastinum is particularly challenging for localization due to its complex anatomical structures and limited accessibility with conventional imaging modalities [3].

Hypercalcemia and hyperparathyroidism are hallmark features of parathyroid adenomas, regardless of their location. However, when these clinical manifestations coincide with inconclusive findings on conventional neck imaging, suspicion of ectopic adenomas should be heightened [4]. In such cases, advanced imaging techniques are crucial for accurate localization and guiding subsequent management strategies [5].

Among advanced imaging modalities, parathyroid scintigraphy is a cornerstone in the diagnostic algorithm for ectopic parathyroid adenomas. With high sensitivity and specificity for detecting abnormal parathyroid tissue, parathyroid scintigraphy provides invaluable insights into the precise anatomical localization of ectopic adenomas, especially in challenging sites like the mediastinum [3]. Additionally, intraoperative parathyroid hormone (PTH) monitoring has emerged as a valuable adjunct, offering real-time assessment of surgical resection adequacy and ensuring the success of parathyroidectomy [6].

Despite these diagnostic advancements, managing ectopic parathyroid adenomas remains complex and multifaceted. Successful treatment necessitates a multidisciplinary approach, involving collaboration among endocrinologists, radiologists, nuclear medicine specialists, and surgeons [5]. Notably, the evolving landscape of minimally invasive surgical techniques, such as robotically assisted thymectomy, offers promising avenues for the effective removal of mediastinal ectopic adenomas while minimizing surgical morbidity.

## Case presentation

A 27-year-old female presented to the hospital with complaints of nausea and vomiting. Two months prior to her admission, she had been diagnosed with hypercalcemia (calcium level: 14 mg/dL; normal range: 8.6-10.2 mg/dL) and elevated PTH levels (PTH: 141 pg/mL; normal range: 15-65 pg/mL). She had received pamidronate treatment, initiated cinacalcet therapy, and was advised to follow up with an endocrinologist, although she had not been able to schedule the appointment.

Initial findings

Upon admission to the hospital, her initial laboratory investigations revealed further elevated serum calcium at 14.7 mg/dL, elevated PTH at 330 pg/mL, a low phosphorus level of 1.6 mg/dL (normal range: 2.7-4.5 mg/dL), and a normal albumin level of 4.4 g/dL (normal range: 3.5-5.3 g/dL). The diagnosis of primary hyperparathyroidism was confirmed; however, attempts to locate the parathyroid adenoma through thyroid ultrasound and computed tomography of the neck were unsuccessful.

Localization

**Figure 1:**
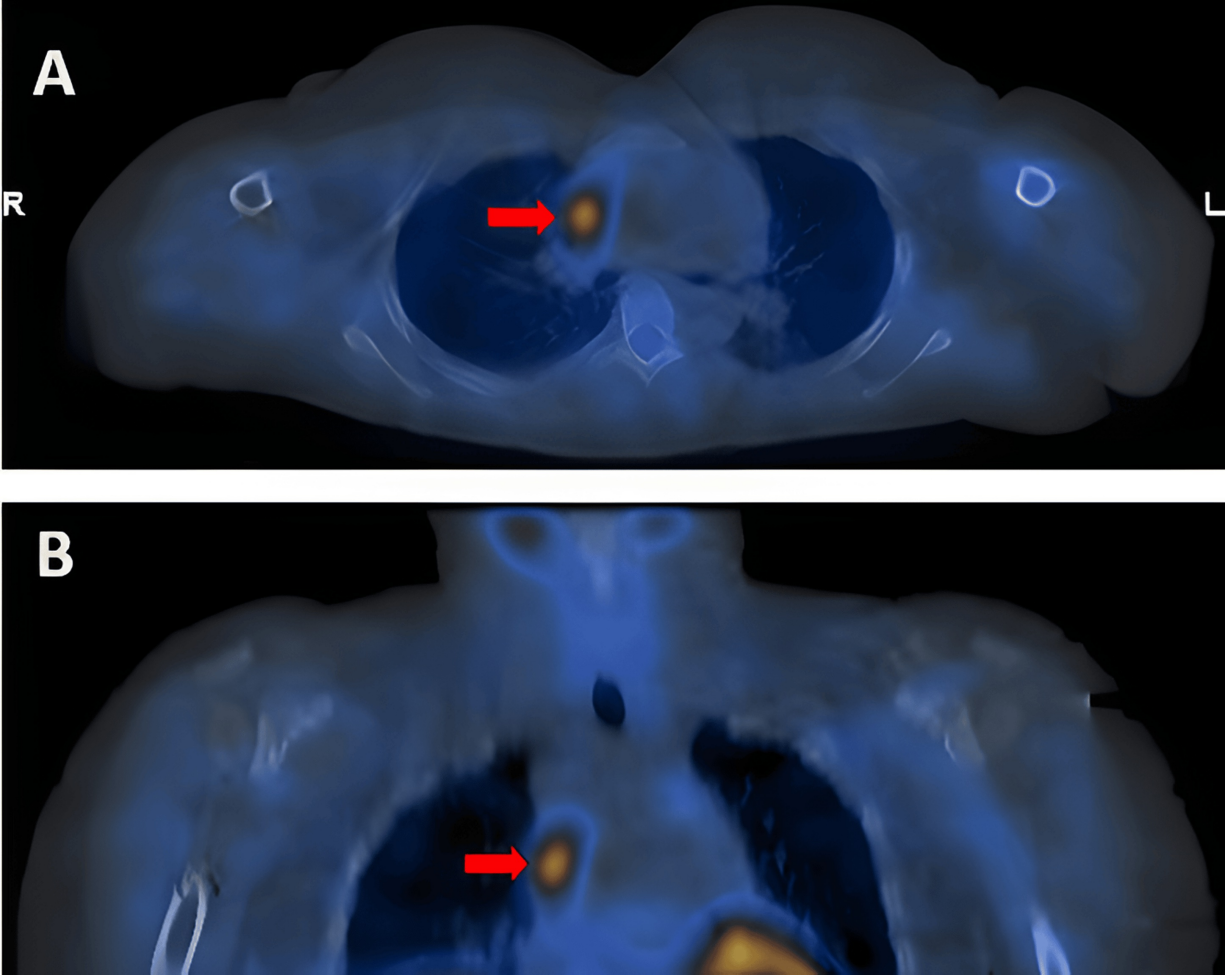
(A) Axial view of technetium-99m sestamibi single photon emission computed tomography/computed tomography. (B) Coronal view of technetium-99m sestamibi single photon emission computed tomography/computed tomography. The red arrow indicates the prominent increased activity in the medial aspect of the right mid to upper chest.

A technetium-99m sestamibi scintigraphy scan identified an ectopic parathyroid adenoma in the medial aspect of the right mid to upper chest (Figure 1). Computed tomography of the chest revealed an oval lesion measuring 1.8 × 0.9 × 2.8 cm in the right anterior mediastinum, corresponding to the site of increased uptake on the technetium sestamibi scintigraphy.

Treatment

**Figure 2:**
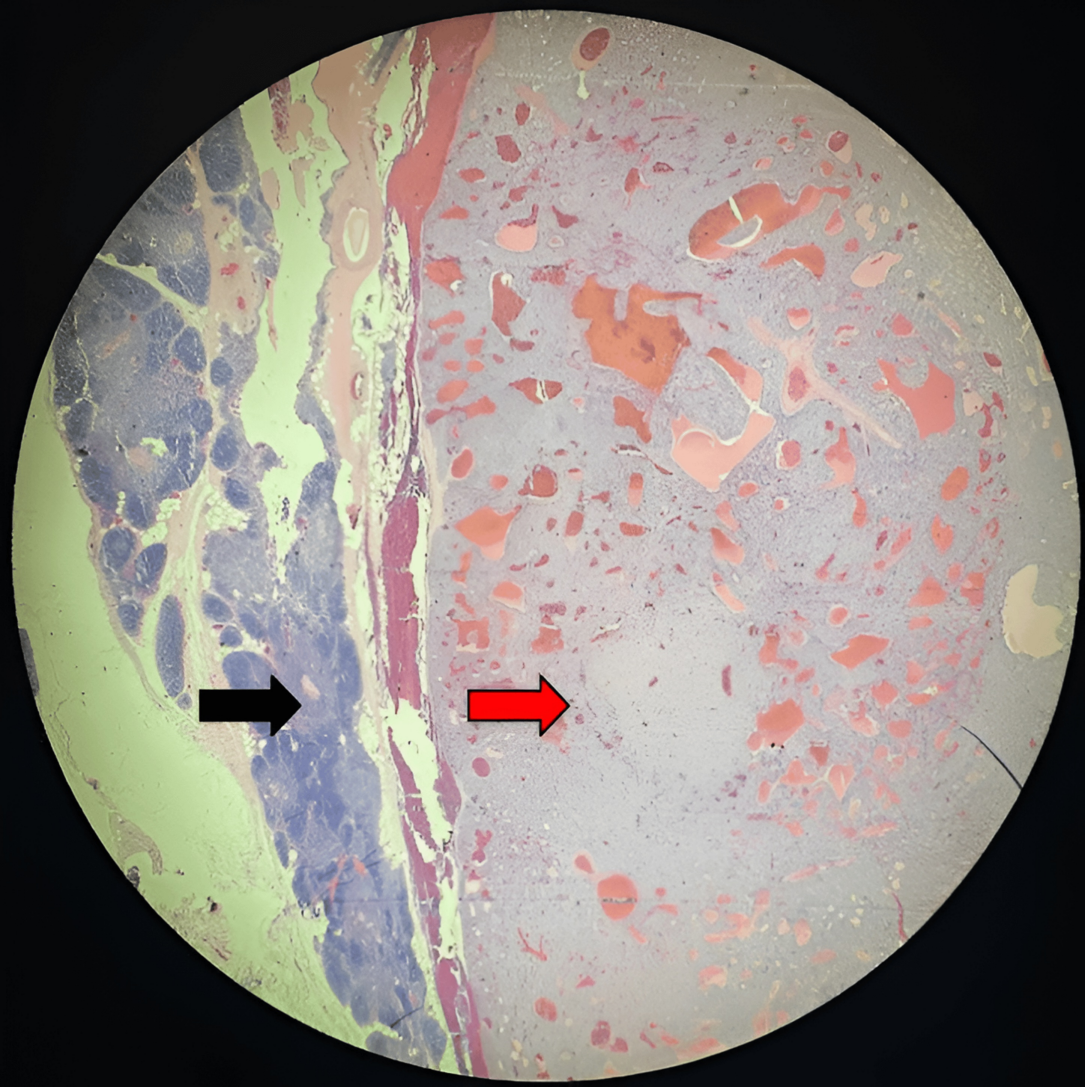
Micropathologic image of the mediastinal mass showing parenchyma composed of lobules separated by adipose tissue, with a dark peripheral cortex and a central clear medulla representing the thymus gland (black arrow). The benign, fairly circumscribed, thinly encapsulated neoplasm, composed of atypical cells arranged in sheets and trabecular patterns separated by thin fibrovascular septae, represents the parathyroid gland (red arrow).

The patient was appropriately hydrated and subsequently transferred to a tertiary medical center, where she underwent a successful robotically assisted thymectomy with intraoperative PTH monitoring. The ectopic adenoma was excised successfully, resulting in the resolution of hypercalcemia (calcium level: 10.3 mg/dL) and normalization of PTH levels (PTH: 18 pg/mL) within one hour after surgery. The diagnosis was confirmed by a postoperative biopsy, which revealed parathyroid tissue within the thymus gland (Figure 2). The patient’s prognosis appears highly favorable, with sustained normalization of serum calcium and PTH levels observed at the eighth-month follow-up.

## Discussion

The constellation of hypercalcemia, elevated PTH levels, hypophosphatemia, and the difficulty in localizing the parathyroid adenoma within the neck strongly suggested an ectopic parathyroid tumor. Diagnosing and managing such cases necessitate a comprehensive approach, often involving advanced imaging techniques to identify and treat elusive mediastinal tumors [7]. Differential diagnoses for mediastinal masses include thymoma, ectopic parathyroid adenoma, and teratoma, among others, emphasizing the complexity of these cases and the need for histopathological confirmation for a definitive diagnosis.

Preoperative imaging is crucial for precise localization and minimally invasive surgery, which can lead to lower complication rates, shorter operating times, reduced hospital stays, and decreased healthcare costs [8]. Although neck ultrasound is typically the initial imaging choice, it has limited sensitivity for detecting ectopic parathyroid glands. Parathyroid scintigraphy offers an 89% sensitivity and a 90% positive predictive value for localizing ectopic glands [9]. Other advanced imaging options include four-dimensional computed tomography, positron emission tomography-computed tomography, magnetic resonance imaging, selective venous sampling, and intraoperative magnetic resonance imaging [8,10].

Our case highlights the importance of integrating clinical findings with advanced imaging modalities such as sestamibi scintigraphy for localizing ectopic parathyroid adenomas [11]. Ectopic parathyroid adenomas are infrequently encountered and can present diagnostic challenges due to their atypical locations. The presence of parathyroid gland tissue within the thymus gland, as confirmed by histopathology in our case (Figure 2), aligns with reported cases in the literature. Previous reports have shown common locations for ectopic glands, with 38% in the thymus and 31% in the retroesophageal region. Cervical ultrasound has a lower sensitivity (59%) and high interoperator variability. Fluorocholine PET/CT is emerging as a promising tool due to its improved spatial resolution and ability to detect smaller adenomas [12]. Four-dimensional computed tomography is increasingly used preoperatively, especially when other imaging modalities are inconclusive, although it is not routinely recommended due to concerns about higher radiation doses and cost.

Surgical removal remains the definitive treatment for ectopic parathyroid adenomas, with options ranging from conventional to minimally invasive techniques. Mediastinal parathyroid adenomas, although rare (approximately 0.8% of cases), require careful surgical planning to avoid complications associated with their complex anatomical locations [13]. This underscores the need for heightened awareness among clinicians and radiologists to consider ectopic parathyroid adenomas in cases of primary hyperparathyroidism with atypical presentations.

## Conclusions

This case report highlights a rare and symptomatic presentation of primary hyperparathyroidism in a young patient caused by an ectopic parathyroid adenoma. Our findings emphasize the diagnostic challenges associated with such cases and stress the importance of considering ectopic locations when initial imaging tests are inconclusive. The successful localization and surgical removal of the ectopic adenoma led to the resolution of the patient’s symptoms and normalization of serum calcium levels. This case underscores the critical role of advanced imaging modalities, such as sestamibi scintigraphy, in facilitating preoperative localization, which enables precise surgical intervention and improves patient outcomes. Further studies are warranted to better understand the prevalence and diagnostic strategies for managing ectopic parathyroid adenomas, especially in young patients with primary hyperparathyroidism.
